# Preparation of Nitrogen-Doped Biochar and Its Adsorption Performance for Cr^6+^ and Pb^2+^ in Aqueous Systems

**DOI:** 10.3390/toxics13050402

**Published:** 2025-05-16

**Authors:** Yazhai Zhang, Zhilei Xia, Aainaa Izyan Nafsun, Weiying Feng

**Affiliations:** 1 Faculty of Chemical and Process Engineering Technology, Universiti Malaysia Pahang Al-Sultan Abdullah, Lebuh Persiaran Tun Khalil Yaakob, Kuantan 26300, Pahang, Malaysia; zhangyazhai06@163.com; 2 College of Chemistry and Chemical Engineering, Hebei Minzu Normal University, Chengde 067000, China; 15732790738@163.com; 3 School of Materials Science and Engineering, Beihang University, Beijing 100191, China

**Keywords:** nitrogen-doped biochar, Cr^6+^ adsorption, Pb^2+^ adsorption, adsorption rate testing, biomass waste utilization

## Abstract

Toxicity and pollution of heavy metals in water environments are very serious threats, and how to efficiently remove heavy metals is a difficult problem in water ecosystems. This study takes Cr and Pb as examples to study the adsorption effects of different types of modified biochar on these two heavy metals and their influencing mechanisms, with the aim of providing precise treatment schemes for water ecological health. Biochar was prepared from apricot branches, apricot shells, and corn stalks through nitrogen doping modification, and its structure and properties were characterized and analyzed. Fourier-transform infrared spectroscopy (FTIR) and scanning electron microscopy (SEM) were employed to investigate the microstructure and surface chemical characteristics of the biochar. Adsorption experiments were conducted to evaluate its removal efficiency for Cr^6+^ and Pb^2+^ from aqueous solutions. The results showed that nitrogen-doped biochar prepared from corn stalks at 600 °C exhibited the highest Cr^6+^ adsorption rate of 81.09%, while the biochar prepared at 500 °C demonstrated the highest Pb^2+^ adsorption rate of 91.61%. Comparative analysis of FTIR and SEM data between nitrogen-doped biochar and its original counterparts revealed the underlying adsorption mechanisms, which involve a synergistic effect of coordination interaction, electrostatic attraction, and chemical reduction. This study highlights nitrogen-doped biochar as an efficient and cost-effective material for the removal of heavy metal ions from aqueous environments. It also provides theoretical and practical insights into the resource utilization of agricultural waste and the management of water pollution.

## 1. Introduction

In recent years, rapid industrialization and urbanization have significantly exacerbated the problem of heavy metal contamination in water systems [1]. Among these pollutants, Cr^6+^ and Pb^2+^ are of particular concern due to their high toxicity, carcinogenicity, and persistent environmental impact [1,2]. Excessive discharge of Cr^6+^ and Pb^2+^ poses severe threats to human health and causes extensive damage to ecosystems [3]. Traditional methods for removing heavy metals from water, such as chemical precipitation and ion exchange, are often costly and challenging to implement on a large scale [4]. Therefore, there is an urgent need for cost-effective, efficient, and environmentally friendly solutions [5]. Biochar derived from the thermochemical conversion of biomass waste has garnered significant research interest due to its cost-effectiveness, renewable nature, and exceptional adsorption capacity for heavy metal remediation. Recent advancements demonstrate the remarkable potential of modified biochar materials in wastewater treatment applications. Shahriyari Far et al. (2021) developed a composite adsorbent combining activated carbon with metal–organic frameworks, achieving a maximum lead ion adsorption capacity of 127 mg/L [6]; similarly, iron-impregnated activated carbon synthesized from coconut shell biomass exhibited adsorption capacities of 11.9 mg/g for Pb^2+^ and 22.1 mg/g for Cr^6+^, respectively [7]. Further enhancement was reported by Abushawish et al. (2022), where nitrogen-doped coconut shell activated carbon demonstrated a maximum Cr^6+^ adsorption capacity of 15.15 mg/g [8].

Biochar, with its high specific surface area, abundant functional groups, and excellent adsorption properties, has emerged as a promising material for heavy metal remediation [9]. Among its variations, nitrogen-doped biochar (N-doped biochar) has shown enhanced adsorption capacity for Cr^6+^ and Pb^2+^ due to the introduction of nitrogen functional groups such as pyridine, pyrrole, and amines. These nitrogen groups not only adsorb heavy metals through coordination and electrostatic attraction but also promote the reduction in Cr^6+^, further improving removal efficiency [10].

For Pb^2+^ removal, studies have demonstrated that amino, pyridinic nitrogen, and pyrrolic nitrogen functional groups on N-doped biochar significantly enhance adsorption capacity through chelation [11]. Bakry et al. reported a maximum Pb^2+^ adsorption capacity of 721 mg/g using nitrogen-doped porous carbon materials (ND-CPC), which surpasses the performance of conventional adsorbents. Moreover, the incorporation of nitrogen functional groups improves the selectivity and stability of the adsorption process, enabling efficient removal of Pb^2+^ even in multi-metal ion systems [11]. Further optimization of the nitrogen doping process has been shown to increase adsorption capacities. For instance, Li et al. found that nitrogen-doped biochar derived from lignocellulosic biomass achieved an adsorption capacity of 1466 mg/g for Pb^2+^, attributed to its abundant microporous structure and high surface nitrogen content [12].

China, as one of the world’s leading corn producers, generates approximately 220 million tons of corn stalks annually, accounting for around 30% of the country’s total crop straw production [13]. In 2019, corn stalks represented 31.45% of the total straw yield from major grain crops in China, making them a vital biomass resource [14]. Corn stalks are primarily concentrated in Northeast, North, and Southwest China, with Heilongjiang, Inner Mongolia, and Henan being major production regions, each exceeding 30 million tons [15]. Despite their potential for energy and material utilization, challenges such as high collection and transportation costs have limited their practical usage [16].

Similarly, apricot branches and shells are by-products of apricot cultivation and processing in regions such as Hebei, Shanxi, and Inner Mongolia, where mountain apricot plantations and almond processing industries are prominent [17]. While detailed production data for apricot branches and shells are limited, Pingquan City in Hebei processes over 3 million kilograms of almonds annually, generating a substantial amount of these by-products [18]. However, these resources are often underutilized and disposed of through burning or landfill, leading to resource waste and environmental pollution [19].

Transforming corn stalks, apricot branches, and apricot shells into nitrogen-doped biochar not only enhances biomass resource utilization but also offers a cost-effective and efficient solution for heavy metal pollution in water systems. This approach aligns with sustainable development principles, delivering significant environmental and economic benefits [20].

This study focuses on typical biomass wastes in China, including corn stalks, apricot branches, and apricot shells, to prepare biochar via nitrogen doping modification. The adsorption performance of the resulting biochars for Cr^6+^ and Pb^2+^ in aqueous solutions is systematically investigated. By comparing the adsorption rates of different biochar types, this research aims to identify highly efficient adsorbents and provide practical solutions for addressing Cr^6+^ and Pb^2+^ contamination in water systems.

## 2. Materials and Methods

### 2.1. Materials

The raw materials used in this study, including corn stalks (JG), apricot branches (XZ), and apricot shells (XK), were sourced from Hebei Province, China. Hebei, located in the North China Plain, is a major producer of corn stalks [21]. Meanwhile, apricot branches and shells are characteristic biomass resources of Hebei’s northern mountainous regions, which are recognized as the largest production area of wild apricot resources in China. These regions generate abundant biomass waste, including apricot branches and apricot shells, on an annual basis. However, these resources remain largely underutilized [18]. The preparation of these abundant raw materials into biochar can not only realize the value-added utilization of biomass waste, but also achieve sustainable water conservation and restoration of the water and soil environment.

### 2.2. Preparation of Nitrogen-Doped Biochar

#### 2.2.1. Preparation of Biomass Raw Materials

Initially, the biomass raw materials were cut into small segments and rinsed thoroughly with clean water to remove surface impurities such as soil. This step ensures the cleanliness of the materials, laying a solid foundation for subsequent processing. After the raw materials are cleaned, they are placed in an oven at 105 °C for 6 h until dry. The dried materials were then ground using a pulverizer until a uniform particle size was achieved, passing through a 40-mesh sieve (Particle size is about 0.35–0.42 μm). The sieved materials were stored for subsequent biochar preparation and adsorption experiments.

#### 2.2.2. Preparation Method of Nitrogen-Doped Biochar

The raw materials sieved in 2.2.1 were impregnated with a 1.5 mol/L ZnCl_2_ solution for 24 h, serving as the activating agent [22]. The activated biomass was then placed in a tubular furnace under a nitrogen atmosphere and heated at a rate of 10 °C/min to 300 °C, 400 °C, 500 °C, and 600 °C, respectively, with a dwell time of 90 min to produce ZnCl_2_-activated biochar (ZnBC).

The activated biochar was subsequently treated with a 15% ammonia/methanol solution and shaken for 60 min. Then, 10 mmol of dicyandiamide was added, and the mixture was stirred for 2 h [23]. The resulting product was transferred into a 1% glutaraldehyde solution for cross-linking, ultrasonicated for 60 min, filtered under vacuum, washed thoroughly, and dried (1 h at 110 °C) until mass stabilization (<0.05% deviation between two consecutive weighings). Finally, the biochar was ground to obtain amine-functionalized activated biochar, referred to as nitrogen-doped activated biochar [24,25] (N-ZnBC).

### 2.3. Characterization of Modified Biochar

#### 2.3.1. Microstructural and Morphological Analysis Using Scanning Electron Microscopy (SEM)

A scanning electron microscope (SEM) (Zeiss Merlin Compact, Oberkochen, Germany) was used to observe the raw materials, including apricot branches, apricot shells, and corn stalks, as well as the nitrogen-doped biochar at magnifications ranging from 500× to 20,000× (×500–×20,000). The morphological differences of biochar before and after nitrogen doping were systematically compared.

#### 2.3.2. Fourier Transform Infrared Spectroscopy (FTIR)

Fourier transform infrared spectroscopy (FTIR) was performed using an FTIR spectrometer (TENSOR 27, BRUKER, Beijing, China) to characterize and analyze the biomass raw materials and nitrogen-doped biochar. The spectral data were used to examine the variations in functional groups before and after nitrogen doping.

#### 2.3.3. Elemental Analysis (CHNS)

The CHNS/O contents were determined using a UNICUBE elemental analyzer (Elementar, Germany) equipped with a thermal conductivity detector (TCD) and direct temperature-programmed desorption (TPD) column. Prior to analysis, solid samples (e.g., graphene, SiC) were degassed under vacuum at customized temperatures (180–300 °C) for 4–8 h to remove adsorbed contaminants. Approximately 4–6 mg of each sample was weighed (±0.01 mg) and combusted at 1200 °C under oxygen-enriched conditions. The gaseous products were separated via TPD technology and quantified by TCD. Calibration was performed using certified reference materials (sulfanilamide and acetanilide), with triplicate measurements showing RSD < 0.1%. Oxygen content was analyzed via high-temperature pyrolysis (1150 °C) with an IR detector module.

#### 2.3.4. Specific Surface Area Analysis (S-BET)

The specific surface area and pore characteristics of biochar samples (G-600, G-300, and Z-500) were determined using a TriStar II Plus 3.03 surface area analyzer (Micromeritics, Norcross, GA, USA) through nitrogen adsorption–desorption isotherms at 77 K. Prior to measurements, each sample underwent vacuum degassing under customized conditions: G-600 at 300 °C for 4 h, G-300 at 180 °C for 8 h, and Z-500 at 250 °C for 5 h, respectively. These pretreatment temperatures were carefully selected below the sample decomposition threshold (>600 °C) to ensure structural integrity while removing physisorbed contaminants.

The Brunauer–Emmett–Teller (BET) method was employed to calculate the specific surface area within the relative pressure range (P/P_0_) of 0.05–0.30, with pore size distributions derived from the adsorption branches using the Barrett–Joyner–Halenda (BJH) model for mesoporous characterization. All measurements were conducted in triplicate using high-purity nitrogen (99.999%) as the adsorbate, with instrument equilibration criteria set at 0.01 mmHg/s pressure tolerance.

### 2.4. Adsorption Experiment

#### 2.4.1. Adsorption Method

The adsorption experiments were conducted using the prepared biochar to assess its capacity to adsorb Cr^6+^ and Pb^2+^ from standard solutions (Cr^6+^ and Pb^2+^). For each experiment, 50 mL of a standard solution was mixed with 50 mg (±2 mg) of biochar. The mixture was stirred continuously for 24 h using a magnetic stirrer. After adsorption, the residual concentration of the solution was measured, and the adsorption efficiency was calculated based on the initial and final concentrations of the standard solutions [26]. Cr^6+^ was prepared using potassium dichromate (K_2_Cr_2_O_7_) and Pb^2+^ using lead nitrate (Pb(NO_3_)_2_). All chemicals were of analytical grade. The initial concentration (c_0_) of Cr^6+^ and Pb^2+^ solutions was 50 mg/L unless otherwise stated. 721UV-Vis spectrophotometer was selected to determine Cr^6+^; Atomic absorption spectrometry was selected for the determination of Pb^2+^.

#### 2.4.2. Adsorption of Chromium

The Cr^6+^ concentration before and after adsorption was determined using a 721 UV-visible spectrophotometer. A standard curve was used to quantify the concentration. The procedure involved using diphenylcarbazide as a chromogenic reagent [27]. First, 0.2000 g of the reagent was weighed into a 250 mL beaker, and 50 mL of acetone was added to dissolve it completely, followed by 50 mL of water for dilution. The solution was mixed thoroughly and set aside for use.

For the adsorption study, 10 mL of the Cr^6+^ solution post-adsorption was diluted to 100 mL. A 20 mL aliquot was then taken and transferred to a 50 mL volumetric flask, to which 3 mL of the chromogenic reagent and 5 mL of dilute sulfuric acid were added. The solution was diluted to the mark with water and allowed to rest for 30 min before measuring absorbance. The concentration before and after adsorption was calculated using the standard curve, and the adsorption efficiency was determined accordingly [28].

#### 2.4.3. Adsorption of Lead

The adsorption of Pb^2+^ was assessed using atomic absorption spectrometry. A standard curve was constructed by measuring the absorbance of Pb^2+^ standard solutions with concentrations of 0, 2, 4, 6, 8, and 10 µg/mL. The absorbance was plotted against the Pb^2+^ concentration to generate the curve. For the adsorption study, the Pb^2+^ solution post-adsorption was diluted tenfold, and its absorbance was measured using an atomic absorption spectrometer. The concentration before and after adsorption was calculated based on the standard curve, and the adsorption efficiency was determined [29]. This method is advantageous due to its simplicity, high accuracy, and excellent sensitivity.

## 3. Results and Discussion

### 3.1. Morphological Analysis by Scanning Electron Microscopy

**Figure 1:**
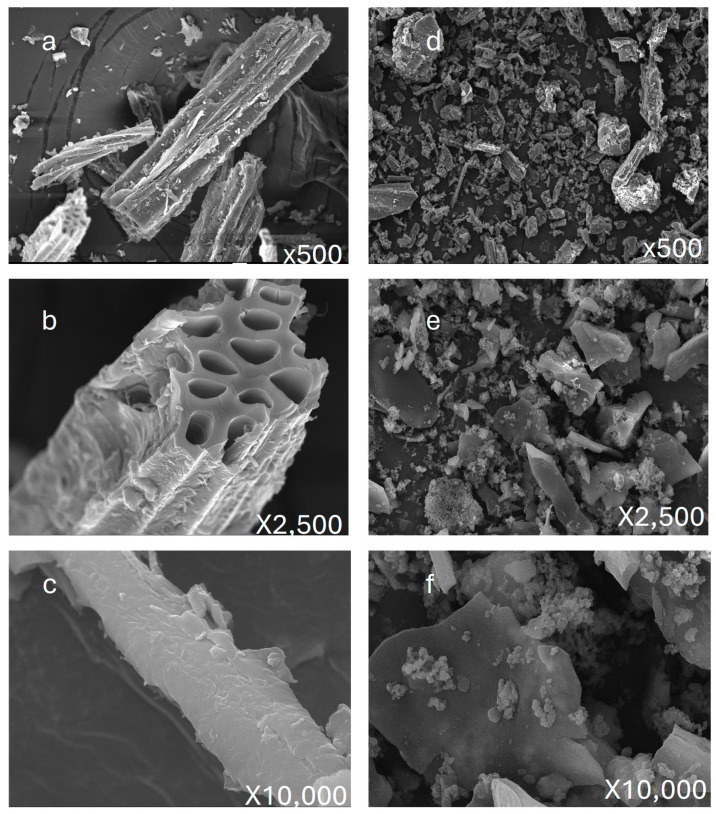
Morphological comparison of biochar before and after nitrogen doping. (**a**–**c**): SEM500×–1000× of biochar made from apricot branches before nitrogen doping and modification (**d**–**f**) Biochar modified by nitrogen doping using apricot branches as raw materials SEM500×–1000×.

Figure 1a–f present a comparative SEM analysis of biochar derived from apricot branches at 500 °C before and after nitrogen doping. The structural evolution induced by nitrogen doping was examined at various magnifications. As shown in Figure 1a–c (500×, 2500×, and 10,000× magnification, respectively), the pristine biochar retains the intrinsic fibrous structure of the raw biomass. The surface morphology exhibits a bundled fiber arrangement with longitudinal grooves (Figure 1a, 500×), a well-defined hollow tubular structure (Figure 1b, 2500×), and a smooth, uniform surface texture (Figure 1c, 10,000×).

Following nitrogen doping, significant morphological transformations are observed at corresponding magnifications (Figure 1d–f). At 500× magnification (Figure 1d), a substantial increase in fine particulate deposition is evident compared to the pristine biochar (Figure 1a). At 2500× magnification (Figure 1e), these particles appear densely packed and unevenly distributed, with an increased presence in the grooves and pores of the fibrous matrix, accompanied by observable agglomeration. Further magnification (Figure 1f, 10,000×) reveals that these particles exhibit irregular shapes, classifying them as amorphous structures. The pronounced formation of amorphous particles significantly enhances the overall specific surface area of the nitrogen-doped biochar, which is expected to influence its physicochemical properties.

**Figure 2:**
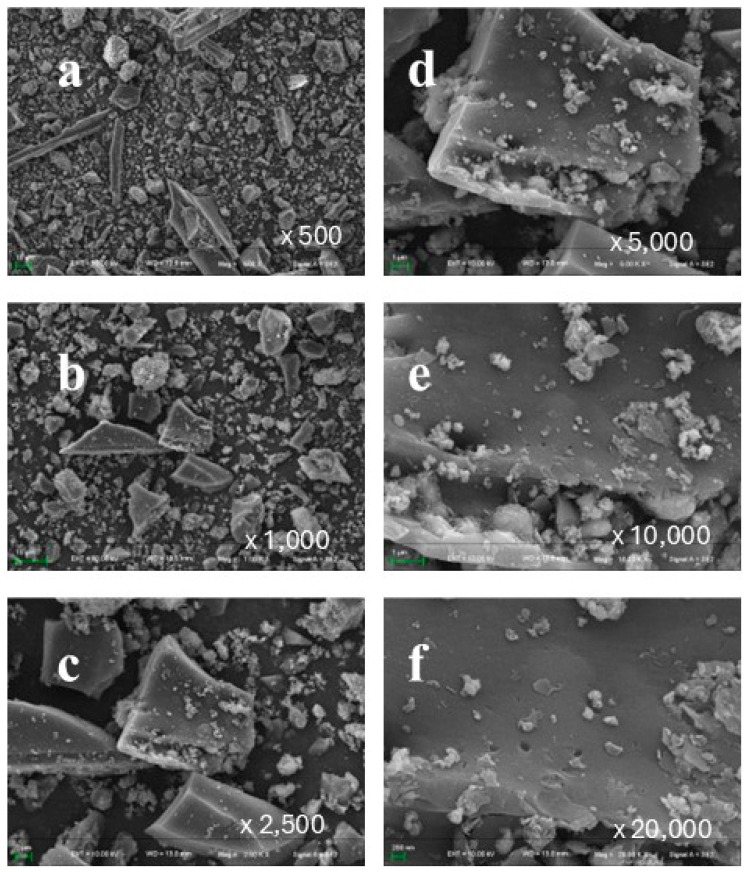
Particle distribution and morphological characteristics of nitrogen-doped biochar. (**a**–**f**) show the morphology of nitrogen-doped biochar made from apricot shell at different magnifications (500×–20,000×).

Figure 2a–f present the distribution and morphological characteristics of nitrogen-doped biochar particles, highlighting the potential correlation between adsorption properties and the type and quantity of surface functional groups. As observed in Figure 2a, the carbonized biomass retains a heterogeneous fibrous matrix, exhibiting structural features such as sheet-like and fragmented fibrous morphologies. These features reflect the intrinsic fiber organization of the biomass as well as the extent of structural disruption induced by the carbonization process. Additionally, numerous fine particles are distributed across the biochar surface, appearing sparsely dispersed on larger fibrous structures while becoming increasingly dense in regions with smaller fiber fragments.

To further examine the morphology of these fine particles, a series of high-magnification SEM images were acquired at different scales: 1000× (Figure 2a), 2000× (Figure 2b), 2500× (Figure 2c), 5000× (Figure 2d), 10,000× (Figure 2e), and 20,000× (Figure 2f). The magnified images reveal that these particles predominantly accumulate within the grooves and porous regions of the fibrous matrix, forming localized agglomerates. Their irregular shape and non-uniform size distribution classify them as amorphous particles.

**Figure 3:**
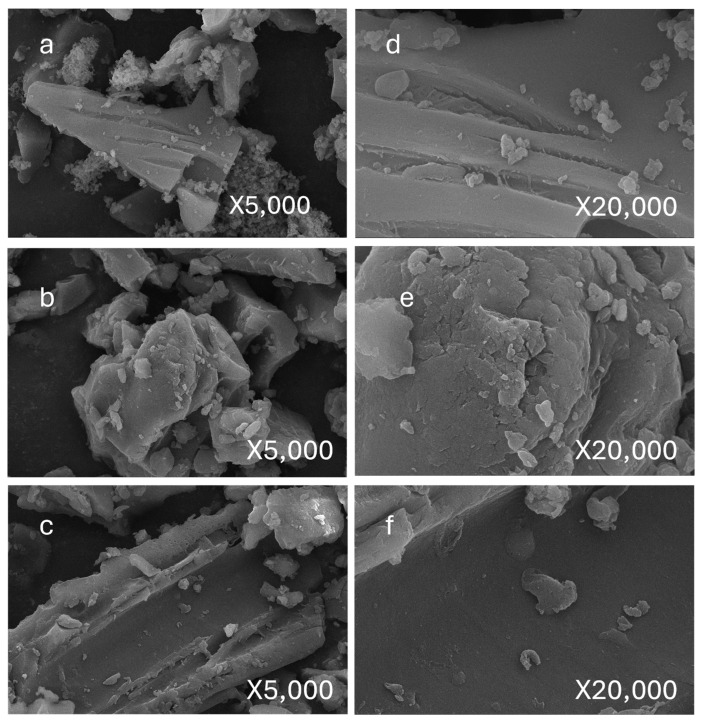
Morphological characteristics of nitrogen-doped biochar derived from different biomass precursors. (**a**): apricot branches as raw materials, nitrogen-doped biochar 5000×, (**b**): apricot shells as raw materials, nitrogen-doped biochar 5000×; (**c**): corn stalks as raw materials, nitrogen-doped biochar 5000×; (**d**): apricot branches as raw materials, nitrogen-doped biochar 20,000×, (**e**): apricot shells as raw materials, nitrogen-doped biochar 20,000×; (**f**): corn stalks as raw materials, nitrogen-doped biochar 20,000×.

Figure 3a–c present SEM images (5000× magnification) of nitrogen-doped biochar derived from apricot branches, apricot shells, and corn stalks, respectively. The results reveal distinct morphological differences among the biomass-derived biochar, attributed to the inherent structural variations of the precursor materials. Despite these differences, all samples exhibit irregularly shaped particles surrounding the fragmented biomass matrix. High-magnification observations of sparsely distributed regions further demonstrate that these fine particles share a smooth surface morphology but lack a defined geometric shape, thus categorizing them as amorphous structures. Note: Figure 3d–f represent the same samples as Figure 3a–c but observed at higher magnification.

**Figure 4:**
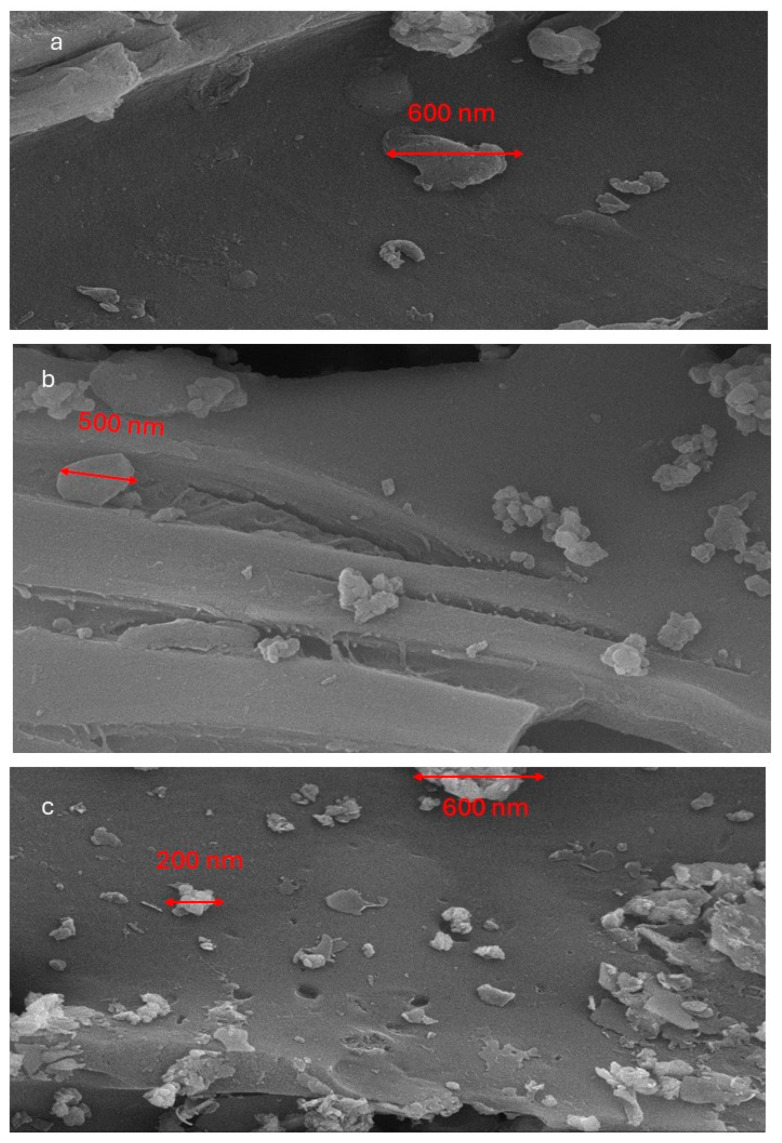
Comparison of amorphous particle sizes of nitrogen-doped biochar derived from different biomass precursors. (**a**): Biochar doped with nitrogen from corn stalks, (**b**): Biochar doped with nitrogen from apricot branches; (**c**): Biochar doped with nitrogen from apricot shells.

Figure 4a–c present high-magnification (20,000×) SEM images of nitrogen-doped biochar derived from corn stalks, apricot branches, and apricot shells, respectively. Particle size analysis was conducted on larger individual particles and agglomeration sites within the images. The results indicate that the maximum observed particle size for nitrogen-doped biochar derived from corn stalks reaches approximately 600 nm, whereas biochar from apricot branches exhibits a maximum particle size of 500 nm. In contrast, the largest particles in nitrogen-doped apricot shell biochar measure around 200 nm, with some agglomerated clusters also reaching 600 nm. These findings suggest that nitrogen-doped biochar derived from different biomass sources predominantly forms amorphous, irregularly shaped particles. However, the size distribution, degree of uniformity, and agglomeration tendency of these amorphous particles are significantly influenced by the intrinsic characteristics of the biomass precursor.

### 3.2. Characterization of Fourier Transform Infrared Spectroscopic

**Figure 5:**
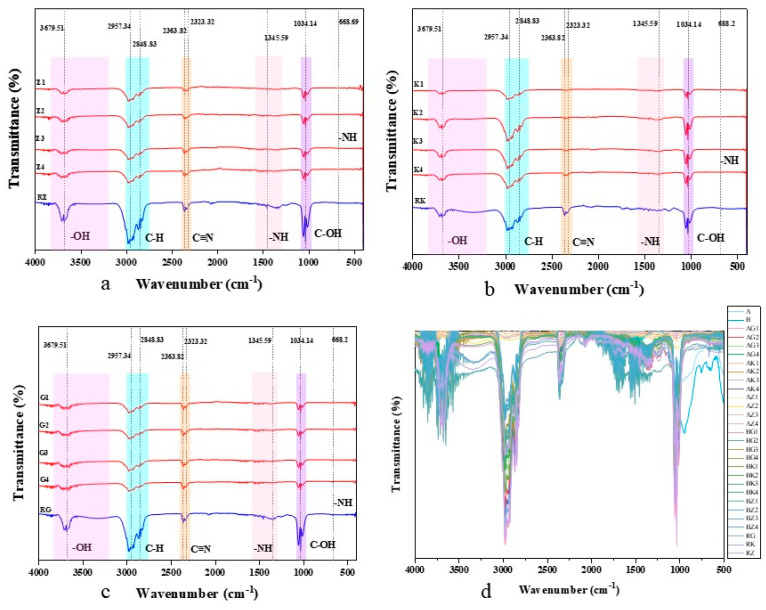
FT-IR of ZnCl_2_-activated N-doped biochars synthesized from different biomass feedstocks at pyrolysis temperatures of 300–600 °C. (**a**) Comparison of infrared spectra of raw material RZ and raw material zinc chloride activated nitrogen modified biochar (Z1–Z4). (**b**) Comparison of infrared spectra of raw material RK and raw material zinc chloride activated nitrogen modified biochar (K1–K4). (**c**) Comparison of infrared spectra of raw material RG and raw material zinc chloride activated nitrogen modified biochar (G1–G4). (**d**) Overlay of FT-IR spectra for all feedstocks (RZ, RK, RG) and their ZnCl_2_-activated N-doped biochars synthesized at four pyrolysis temperatures (Z1–Z4, K1–K4, G1–G4). Notes: Sample nomenclature: Subscripts denote pyrolysis temperatures (1: 300 °C, 2: 400 °C, 3: 500 °C, 4: 600 °C).

FT-IR spectroscopy is an effective tool for identifying surface functional groups on biochar. The infrared spectra of the 12 samples analyzed were generally similar (Figure 5). The spectrum reveals characteristic peaks at 3697.51 cm^−1^, corresponding to the stretching vibration of –OH bonds, at approximately 2957.34 cm^−1^ for C-H bonds, around 2363.82 and 2323.32 cm^−1^ for C≡N bonds, and at 1345.59 cm^−1^, indicating the presence of –NH bonds, and at 1034.14 cm^−1^, indicating the presence of C–OH bonds, in the biochar [30].

As nitrogen-doped biochar was prepared, the presence of C≡N, N–H bonds confirms the existence of active functional groups on the biochar surface [31]. During the adsorption process, the lone pair of electrons in amino groups can interact with certain heavy metal ions [32]. For instance, Pb^2+^ can form coordination complexes with amino groups, and amino groups with reducing properties can serve as electron donors, directly reducing Cr^6+^ to Cr^3+^ [32]. Thus, the presence of amino groups enables biochar to undergo not only physical adsorption but also chemical adsorption during the adsorption process [33].

### 3.3. Elemental Analysis

Elemental analysis was conducted on three nitrogen-doped biochars—G-600 (corn straw-derived, pyrolyzed at 600 °C, characterized by high adsorption performance), G-300 (corn straw-derived, 300 °C, low adsorption performance), and Z-500 (apricot branch-derived, 500 °C, the best among its type but inferior to G-600)—to elucidate their adsorption behavior toward Cr^6+^ and Pb^2+^.

**Figure 6:**
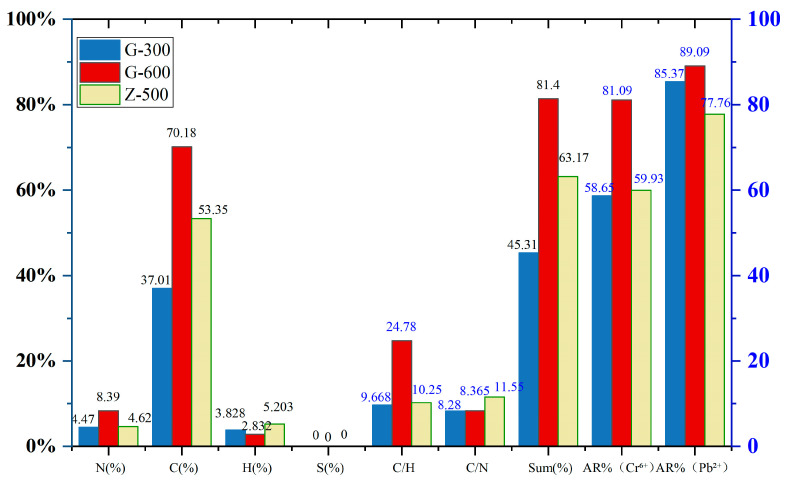
Comparative analysis of the distribution of basic elements such as CHN and the adsorption rate of Cr^6+^ and Pb^2+^. by biochar.

To explore the influence of pyrolysis temperature and biomass type, comparisons were made between G-600 and G-300 (same biomass, different temperatures), and between G-600 and Z-500 (different biomass, both with relatively high adsorption). The elemental composition (C, H, N, S) was determined for each sample, and relevant parameters including C/H, C/N ratios, and the total elemental proportion (Sum%, the combined wt.% of C, H, N, and S) were calculated. These data were then correlated with the adsorption removal efficiencies (AR%) of Cr^6+^ and Pb^2+^, as illustrated in Figure 6.

The results demonstrated that C%, N%, C/H ratio, and Sum% showed trends consistent with the AR% values of both Cr^6+^ and Pb^2+^. A higher C% and C/H ratio reflect a greater degree of carbonization, indicating that increased carbonization enhances adsorption performance. Similarly, elevated N% corresponds to higher nitrogen doping levels, which also contribute positively to adsorption capacity. In all three biochars, the combined content of C, H, N, and S was significantly below 100%, suggesting the presence of ash or inorganic constituents. The lower the Sum% value, the more anionic residues (e.g., negatively charged species) may be inferred. These anions are presumed to enhance electrostatic attraction toward positively charged Cr^6+^ and Pb^2+^ ions.

Interestingly, despite theoretical expectations that electrostatic interactions should favor Cr^6+^ adsorption over Pb^2+^, the experimental results showed stronger Pb^2+^ removal. This indicates that other factors, such as the degree of carbonization or nitrogen functionalization, may play a more dominant role than electrostatic interactions in this system.

Comparative analysis of G-600 vs. G-300 and G-600 vs. Z-500 revealed an inverse correlation between H% and AR% for both Cr^6+^ and Pb^2+^. Since H% indirectly reflects the abundance of H-containing functional groups such as –OH and –NH_2_, this finding suggests that these groups did not significantly contribute to enhanced adsorption in this study. Although the C/N ratio followed the same trend as the AR% values, the difference in C/N between G-600 and G-300 (same feedstock) was minimal, indicating that pyrolysis temperature had limited impact on nitrogen incorporation. In contrast, the C/N ratio differed substantially between G-600 (corn straw) and Z-500 (apricot branch), highlighting that nitrogen doping efficiency is more sensitive to feedstock type than to pyrolysis temperature.

### 3.4. Specific Surface Area

The relevant data are summarized in Table 1 according to the analysis and test report

The results of the analysis are as follows:

(1). BET Surface Area G (Corn straw) > Z (apricot branch): the specific surface area of G-300 (raw material: corn stalk, biochar preparation temperature 300 °C)and G-600 (raw material: corn stalk, biochar preparation temperature 600 °C) is much higher than that of Z-500 (raw material: apricot branch, biochar preparation temperature 500 °C). It is shown that corn straw is more likely to form a developed pore structure in the roasting process, especially when it is not excessively sintered at 300 °C.

(2) Pore volume

The G-300 (300 °C) has a significantly higher total pore volume (0.045 cm^3^/g), possibly due to the retention of more large mesopores or incomplete release of some volatiles, resulting in more pores.

(3) Percentage of micropores

The G-600 has a higher proportion of micropores (0.00305 cm^3^/g), indicating that the increase in roasting temperature is conducive to the generation of more micropores. Micropores are the key to adsorption capacity and are suitable for small molecules. In contrast, although G-300 has the highest specific surface area, it contributes little to micropores, indicating that it is mainly composed of medium and large pores.

(4) Average Pore Size

The average pore size of G-300 is the largest (nearly 14 nm), indicating that it is dominated by large mesopores; the average pore size of G-600 and Z-500 is 4–7 nm, which belongs to small mesopores.

(5) Comparative analysis of G-600 and G-300

Characteristics and adsorption advantages of G-600: the proportion of micropores is large (about 62%), which is conducive to enhancing the adsorption capacity; the pore size is smaller (4.66 nm), which is suitable for adsorption of small molecules;

(6) Characteristics and adsorption advantages of G-300:The specific surface area is slightly higher, but most of it is contributed by medium and large pores; the pore volume is large (0.045 cm^3^/g), which is beneficial to the adsorption of large molecules; the pore size is large (13.99 nm), which is conducive to rapid diffusion, but the adsorption force may be insufficient for small molecules.

(7) Summary

Corn stalk biochar calcined at 300 °C exhibited the highest BET surface area (12.76 m^2^/g) and a significantly larger total pore volume (0.045 cm^3^/g), with an average pore diameter of ~14 nm. This meso-to-macroporous structure is better suited for the adsorption of large molecules but limited micropore content may reduce the affinity for small molecule adsorption. In contrast, the 600 °C sample developed more micropores (micropore area accounting for ~62% of BET area) with a smaller average pore diameter of 4.66 nm. It leads to a higher micropore contribution and better small molecule absorption. The apricot branch biochar calcined at 500 °C showed a much lower surface area (3.70 m^2^/g) and pore volume (0.006 cm^3^/g). It suggests a relatively underdeveloped pore structure after thermal treatment.

### 3.5. Removal Efficiency of Cr^6+^ and Pb^2+^

Biochar was prepared from apricot branches, apricot shells, and corn stalks at pyrolysis temperatures of 300 °C, 400 °C, 500 °C, and 600 °C, followed by nitrogen doping. The resulting nitrogen-doped carbon materials were denoted as N-XZ-ZnBC-T (300 °C/400 °C/500 °C/600 °C). The removal efficiency of Cr^6+^ and Pb^2+^ from aqueous solutions was evaluated based on the adsorption rate, where a higher adsorption rate indicates superior removal performance.

#### 3.5.1. Removal Efficiency of Cr^6+^

**Figure 7:**
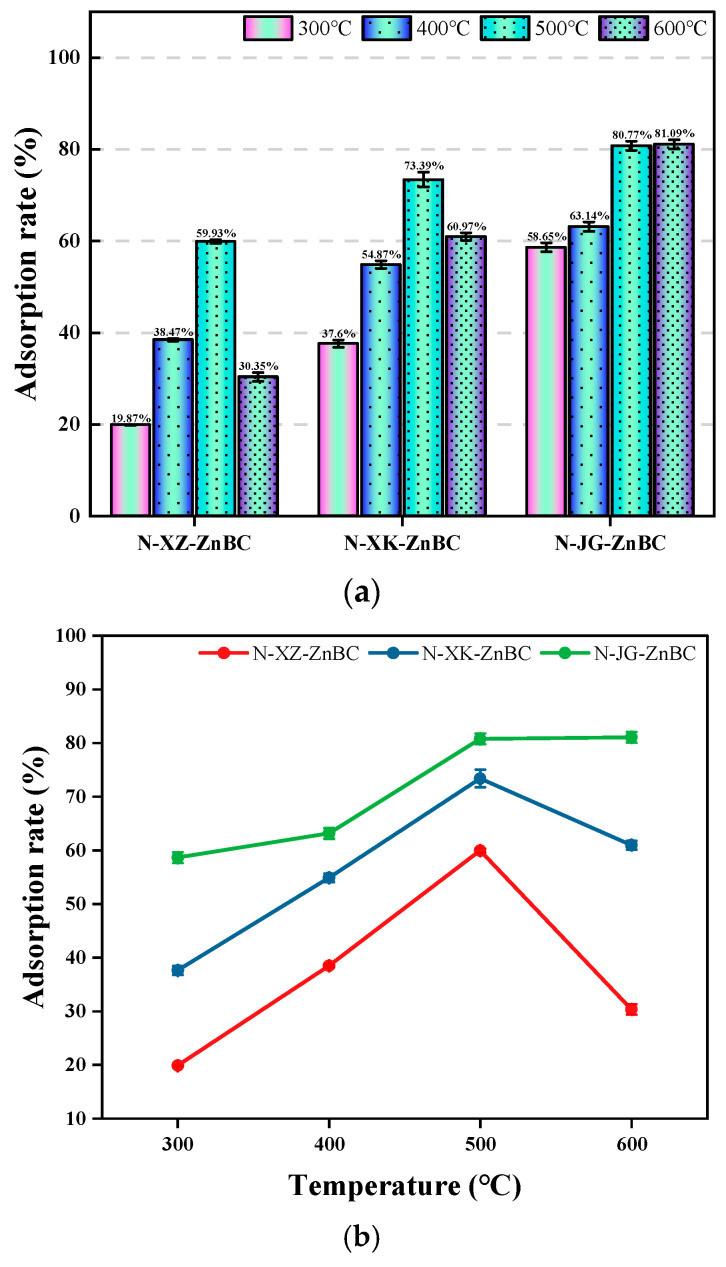
(**a**) Adsorption rate of Cr^6+^ by N-ZnBC at different temperatures for each raw material; (**b**) Effect of temperature on Cr^6+^ adsorption efficiency of carbon materials.

The concentration and absorbance of Cr^6+^ before and after adsorption experiments were measured, and the adsorption rate was calculated using Equation ($\left({AR}_{{Cr}^{6+}}=\frac{C_{0}-C_{A}}{C_{0}}\times 100\%\right)$),$\;AC\left(Adsorption\;capacity\right)=\frac{{(C}_{0}*AR/1000)*50}{50\mathrm{mg}*10^{-3}}\left(\frac{\mathrm{mg}}{g}\right)$. Figure 7 presents the statistical analysis of Cr^6+^ removal efficiency for the twelve biochar samples. As shown in Figure 7a, only N-JG-ZnBC-T (500 °C/600 °C) achieved an adsorption rate exceeding 80%. Figure 7b illustrates the relationship between pyrolysis temperature and adsorption efficiency, revealing that nitrogen-doped biochar derived from corn stalks exhibited the highest Cr^6+^ adsorption rate of 81.09% at 600 °C, followed closely by an adsorption rate of 80.77% at 500 °C. Given that both rates are approximately 81% (AC40.5mg/g), the 500 °C condition is considered the optimal choice in terms of energy efficiency. The adsorption rate of both N-XZ-ZnBC and N-XK-ZnBC increased with pyrolysis temperature up to 500 °C, after which a decline was observed. Therefore, 500 °C was identified as the optimal pyrolysis temperature for the preparation of nitrogen-doped biochars N-XZ-ZnBC and N-XK-ZnBC. Among the three biomass feedstocks, nitrogen-doped biochar from corn stalks demonstrated the highest Cr^6+^ removal efficiency, followed by apricot shells, while apricot branches exhibited the lowest performance.

#### 3.5.2. Removal Efficiency of Pb^2+^

**Figure 8:**
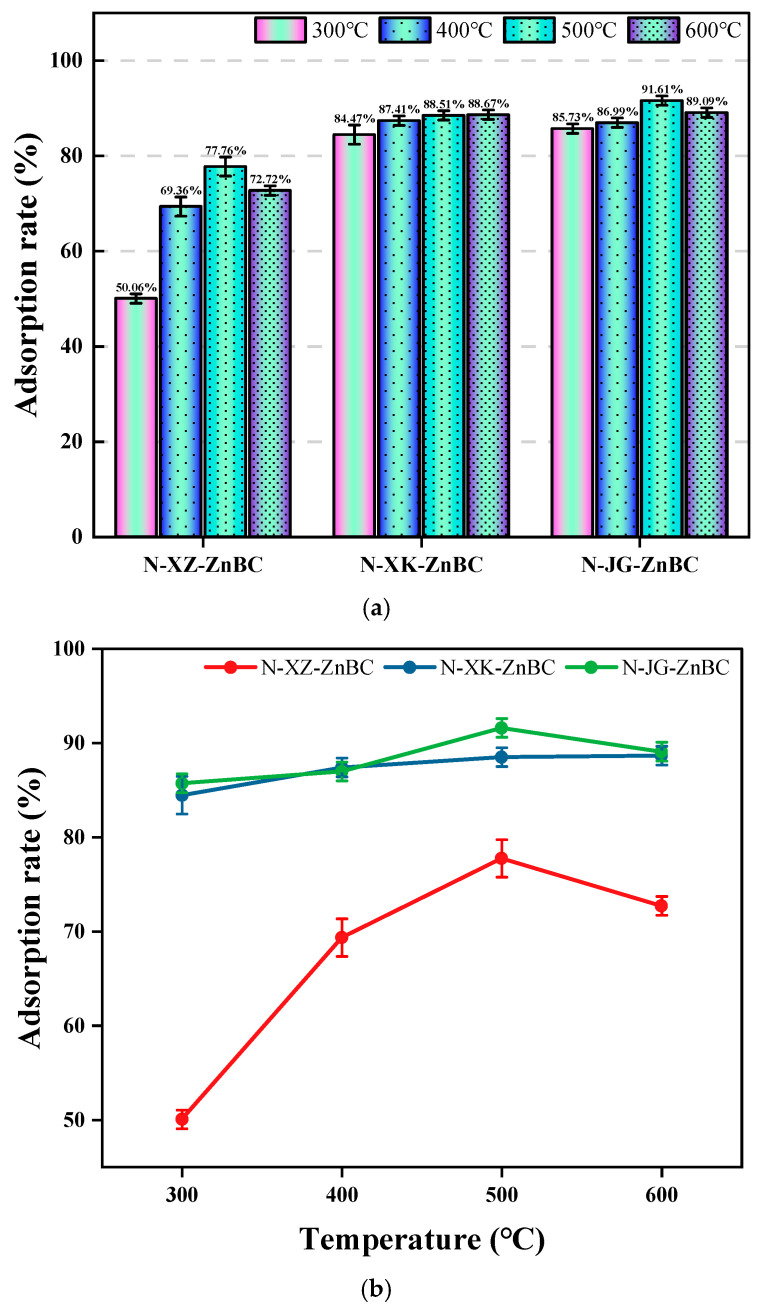
(**a**) Adsorption rate of Pb^2+^ by N-ZnBC at different temperatures for each raw material; (**b**) Effect of temperature on Pb^2+^ adsorption efficiency of carbon materials.

The concentration and absorbance of Pb^2+^ before and after the adsorption experiments were measured, and the adsorption rate was calculated using equation $\left({AR}_{{Pb}^{2+}}=\frac{{12[C}_{0}-C_{A}]}{C_{0}}\times 100\%\right)$. $13[AC\left(Adsorption\;capacity\right)=\frac{{(C}_{0}*AR/1000)*50}{50\mathrm{mg}*10^{-3}}\left(\frac{\mathrm{mg}}{g}\right)]$. Figure 8 presents the statistical analysis of Pb^2+^ removal efficiency for the twelve biochar samples.

As shown in Figure 8a, eight nitrogen-doped biochar samples, including N-XK-ZnBC-T (300 °C/400 °C/500 °C/600 °C) and N-JG-ZnBC-T (300 °C/400 °C/500 °C/600 °C), exhibited consistently high Pb^2+^ removal efficiency, with adsorption rates ranging from a minimum of 84.47% to a maximum of 91.61%(AC-45.81 mg/g). This result suggests that nitrogen-doped biochar derived from apricot shells and corn stalks serves as an effective adsorbent for Pb^2+^ removal from aqueous solutions. Notably, these materials maintained high adsorption efficiency even at lower pyrolysis temperatures.

Figure 8b illustrates the effect of temperature on the adsorption performance of nitrogen-doped biochar derived from the three biomass feedstocks. The results indicate that, for all three feedstocks, biochar prepared at 500 °C achieved the highest adsorption rate. Among them, corn stalk-derived biochar exhibited the best Pb^2+^ adsorption performance, followed by apricot shells, while apricot branches performed the worst. Additionally, nitrogen-doped biochar from apricot branches did not achieve an adsorption rate exceeding 80% under any pyrolysis temperature. In summary, Figure 8 highlights that corn stalks and apricot shells are the most suitable biomass feedstocks for producing nitrogen-doped biochar with high Pb^2+^ removal efficiency. Even at a low pyrolysis temperature of 300 °C, biochar derived from these materials demonstrated effective Pb^2+^ adsorption performance.

Apricot shell, apricot branch, and corn stalk were selected as raw materials to prepare biochar. The adsorption performance of biochar varies depending on the raw material [34]. A comparison of these results indicates that biochar derived from apricot shell (XK-N-ZnBC) and corn stalk (JG-N-ZnBC) consistently demonstrated high Pb^2+^ adsorption rates (>84.47%) across all temperatures (300–600 °C), with similar overall performance for both raw materials. In contrast, biochar derived from apricot branch (XZ-N-ZnBC) showed significantly lower Pb^2+^ adsorption rates, with a maximum value of 77.76%.

For Cr^6+^ adsorption, only 600 °C-JG-N-ZnBC and 500 °C-JG-N-ZnBC exhibited relatively high adsorption rates of 81.09% and 80.77%, respectively. Biochars derived from apricot shell and apricot branch under all temperature conditions exhibited comparatively low Cr^6+^ adsorption rates. In summary, the selection of raw materials for biochar preparation should consider both the type of target ion and the availability of agricultural residues in different regions. For Cr^6+^ adsorption, corn stalk is recommended as the preferred raw material. For Pb^2+^ adsorption, regions like Chengde that produce abundant apricots can utilize apricot shells, while regions like Northeast China, rich in corn production, can opt for corn stalks as a biomass source.

### 3.6. Effect of Adsorption Time on Adsorption Performance

**Figure 9:**
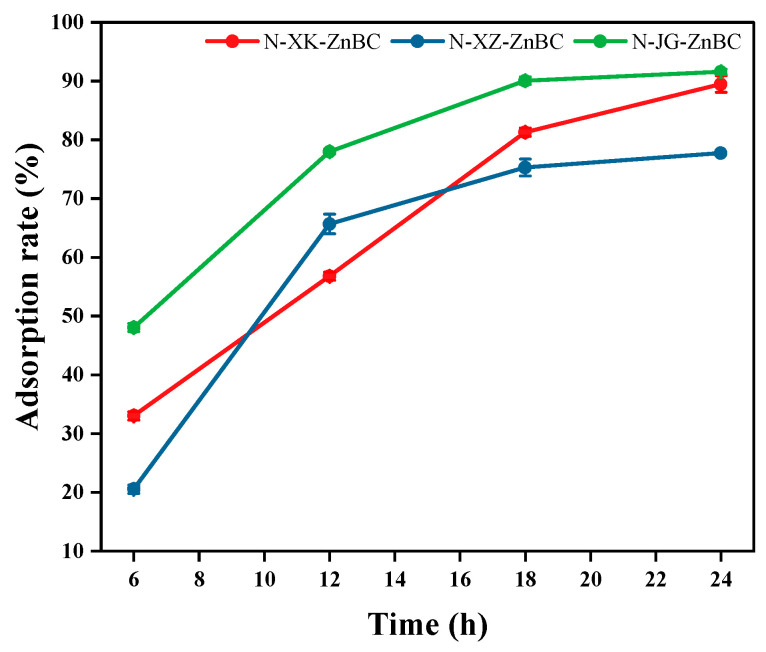
Adsorption rate for different time periods.

To explore the adsorption rates at various time intervals, nitrogen-doped biochar prepared from three biomass feedstocks was tested at four time points: 6, 12, 18, and 24 h. Taking the adsorption of lead ions by biochar prepared at 500 °C as an example, the adsorption rates for apricot shell biochar were 33.02%, 56.79%, 81.32%, and 89.51% at the respective time intervals. For apricot branch biochar, the adsorption rates were 20.55%, 65.68%, 75.30%, and 77.76%. Corn stalk biochar exhibited adsorption rates of 48.06%, 77.96%, 90.06%, and 91.61% over the same time periods. The line chart illustrating the adsorption rates over time is shown in Figure 9.

Based on Figure 9, it can be observed that the adsorption rates of biochar derived from the three biomass feedstocks increased rapidly during the 6–12 h interval and then gradually approached equilibrium. Specifically, the adsorption rate for apricot shell biochar exhibited minimal growth between 18–24 h, reaching equilibrium after 18 h. For apricot branch biochar, the growth rate began to decrease after 12 h, achieving equilibrium within 24 h. Corn stalk biochar demonstrated a growth of less than 2% between 18–24 h, indicating that equilibrium was already achieved during this period. These findings confirm that the selected 24-h adsorption equilibrium time for the biochars is scientifically valid and reliable.

## 4. Conclusions

Nitrogen-doped biochar was successfully synthesized and evaluated for its adsorption performance toward Pb^2+^ and Cr^6+^. The nitrogen-doped biochar exhibited significantly higher adsorption efficiency for Pb^2+^ compared to Cr^6+^. FT-IR analysis indicated the introduction of -NH_2_ groups in the nitrogen-doped biochar, which can interact with heavy metals. The adsorption process involves both physical and chemical adsorption, the latter enhancing the overall adsorption capacity.

SEM images revealed the presence of numerous pores of varying sizes and shapes on the biochar surface. Post-adsorption, these pores were found to contain a substantial number of particles, confirming their role in the physical adsorption of heavy metal ions, thus improving adsorption performance. Among the eight nitrogen-doped biochars derived from apricot shells and corn stalks (activated at 300–600 °C), the materials showed high adsorption efficiency for Pb^2+^, with adsorption rates exceeding 84.47%. The highest adsorption rates were observed for 500 °C-XK-N-ZnBC (89.51%) and 500 °C-JG-N-ZnBC (91.61%). The obtained adsorption capacity (45.81 mg/g) is significantly higher than that of recently reported modified coconut shell-derived carbon for Pb^2+^ removal (11.9 mg/g) [7].

For Cr^6+^ adsorption, only two biochars—600 °C-JG-N-ZnBC (81.09%) and 500 °C-JG-N-ZnBC (80.77%)—demonstrated relatively high efficiencies. The adsorption capacity achieved in this study (40.5 mg/g) is substantially higher than those reported for Cr^6+^ removal using modified coconut shell biochar (22.1 mg/g) [7] and nitrogen-doped coconut shell activated carbon (15.15 mg/g) [8], respectively.

For Cr^6+^ adsorption, only two biochars—600 °C-JG-N-ZnBC (81.09%) and 500 °C-JG-N-ZnBC (80.77%)—demonstrated relatively high efficiencies. Future studies should systematically investigate the influence of pH on Cr^6+^ adsorption. Additionally, improvements in testing methods for equilibrium adsorption capacity and adsorption rate could further refine the findings. While this study primarily focused on the performance evaluation of nitrogen-doped biochars for the removal of Cr^6+^ and Pb^2+^ from aqueous solutions, future work should include a techno-economic assessment to determine the feasibility of a large-scale application. The utilization of biomass waste not only adds value to agricultural residues but also contributes to environmental sustainability. Moreover, the incorporation of nitrogen into the biochar structure offers potential agronomic benefits when applied to soil, such as acting as a slow-release nutrient and reducing the risk of secondary pollution. Therefore, evaluating both the environmental and economic implications of this approach is essential to support its practical deployment in water and soil remediation systems.

## Figures and Tables

**Table 1:** The surface test results of three typical nitrogen-doped biochar ratios were compared with the adsorption rates of Cr^6+^ and Pb^2+^.

Parameter	G-300	G-600	Z-500
BET Surface Area (m^2^/g)	12.76	10.95	3.697
Langmuir Surface Area (m^2^/g)	13.79	10.14	3.606
Micropore Area (t-Plot, m^2^/g)	3.180	6.819	2.109
External Surface Area (m^2^/g)	9.578	4.130	1.587
Total Pore Volume (cm^3^/g)	0.04461	0.01275	0.005997
Micropore Volume (cm^3^/g)	0.001291	0.003048	0.000908
Average Pore Diameter (nm, BET)	13.99	4.656	6.489
AR%Cr(VI)	58.65	81.09	59.93
AR%Pb(II)	85.73	89.09	77.76

## Data Availability

The data are already in the article.
